# Preclinical Advances in Theranostics for the Different Molecular Subtypes of Breast Cancer

**DOI:** 10.3389/fphar.2021.627693

**Published:** 2021-04-27

**Authors:** Hanyi Fang, Alessandra Cavaliere, Ziqi Li, Yiyun Huang, Bernadette Marquez-Nostra

**Affiliations:** ^1^ PET Center, Department of Radiology and Biomedical Imaging, School of Medicine, Yale University, New Haven, CT, United States; ^2^ Department of Nuclear Medicine, Union Hospital, Tongji Medical College, Huazhong University of Science and Technology, Wuhan, China; ^3^ Hubei Province Key Laboratory of Molecular Imaging, Wuhan, China; ^4^ Department of Nuclear Medicine, Tongji Hospital, Tongji Medical College, Huazhong University of Science and Technology, Wuhan, China

**Keywords:** theranostics (combined therapeutic and diagnostic technology), breast cancer subtypes, molecular imaging, targeted therapy, preclinical (*in-vivo*) studies, positron emission tomography, single-photon emission computed tomography

## Abstract

Breast cancer is the most common cancer in women worldwide. The heterogeneity of breast cancer and drug resistance to therapies make the diagnosis and treatment difficult. Molecular imaging methods with positron emission tomography (PET) and single-photon emission tomography (SPECT) provide useful tools to diagnose, predict, and monitor the response of therapy, contributing to precision medicine for breast cancer patients. Recently, many efforts have been made to find new targets for breast cancer therapy to overcome resistance to standard of care treatments, giving rise to new therapeutic agents to offer more options for patients with breast cancer. The combination of diagnostic and therapeutic strategies forms the foundation of theranostics. Some of these theranostic agents exhibit high potential to be translated to clinic. In this review, we highlight the most recent advances in theranostics of the different molecular subtypes of breast cancer in preclinical studies.

## Introduction

The molecular subtypes of breast cancer are classified based on the status of estrogen receptor (ER), progesterone receptor (PR), and human epidermal growth factor receptor 2 (HER2). In addition to surgery, radiotherapy, and chemotherapy, the standard of care targeted treatments for breast cancer vary according to the molecular subtype of patient’s tumor and stage of the disease. For ER-positive breast cancer, ER-targeting tamoxifen is the primary therapy for the non-metastastic luminal subtype of breast cancer (Masoud and Pagès, 2017). For HER2-positive breast cancer, monoclonal antibodies targeting this receptor, such as trastuzumab, have greatly improved the survival of breast cancer patients with non-metastatic disease (Waks and Winer, 2019). Finally, patients with advanced triple negative breast cancer are recently benefiting from immune therapy with the FDA-approval of the monoclonal antibody atezolizumab (Cyprian et al., 2019) or from the antibody drug conjugate, sacituzumab govitecan (Bardia et al., 2019).

However, patients with any breast cancer subtype can have intrinsic or acquired resistance to different therapeutics including chemotherapy, endocrine therapy, and targeted therapy (Chun et al., 2017). Further, tumor heterogeneity in the same patient presents a challenge for achieving complete and durable responses to targeted treatments (Steding, 2016; Blasco-Benito et al., 2018). To overcome resistance to standard of care treatments, many efforts have turned to combination therapies or addition of toxic payloads, such as ionizing radiation, for targeted radionuclide therapy (TRT) (Lopez and Banerji, 2017). Many investigations have also been devoted to find new therapeutic targets closely associated with breast cancer aggressiveness, invasion, metastasis, and recurrence, such the gastrin-releasing peptide receptors (GRPR) overexpressed on the surface of cancer cells, cyclin-dependent kinase 4 and 6 (CDK4/6), histone deacetylase (HDACs), and *MYC* proto-oncogene. The availability of new targeted treatments for these targets prompted the development of non-invasive imaging agents via positron emission tomography (PET) or single-photon emission tomography (SPECT) to help select patients most likely to benefit from these treatments. The combination of diagnostic imaging and therapy forms the foundation of theranostics.

**FIGURE 1:**
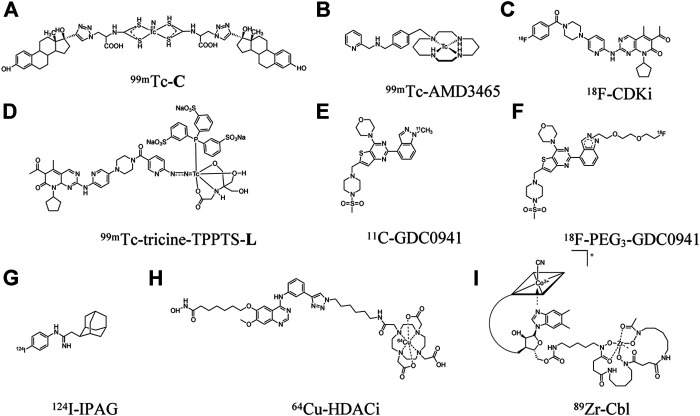
Structures of radiolabeled small molecules for imaging of breast cancer.

**FIGURE 2:**
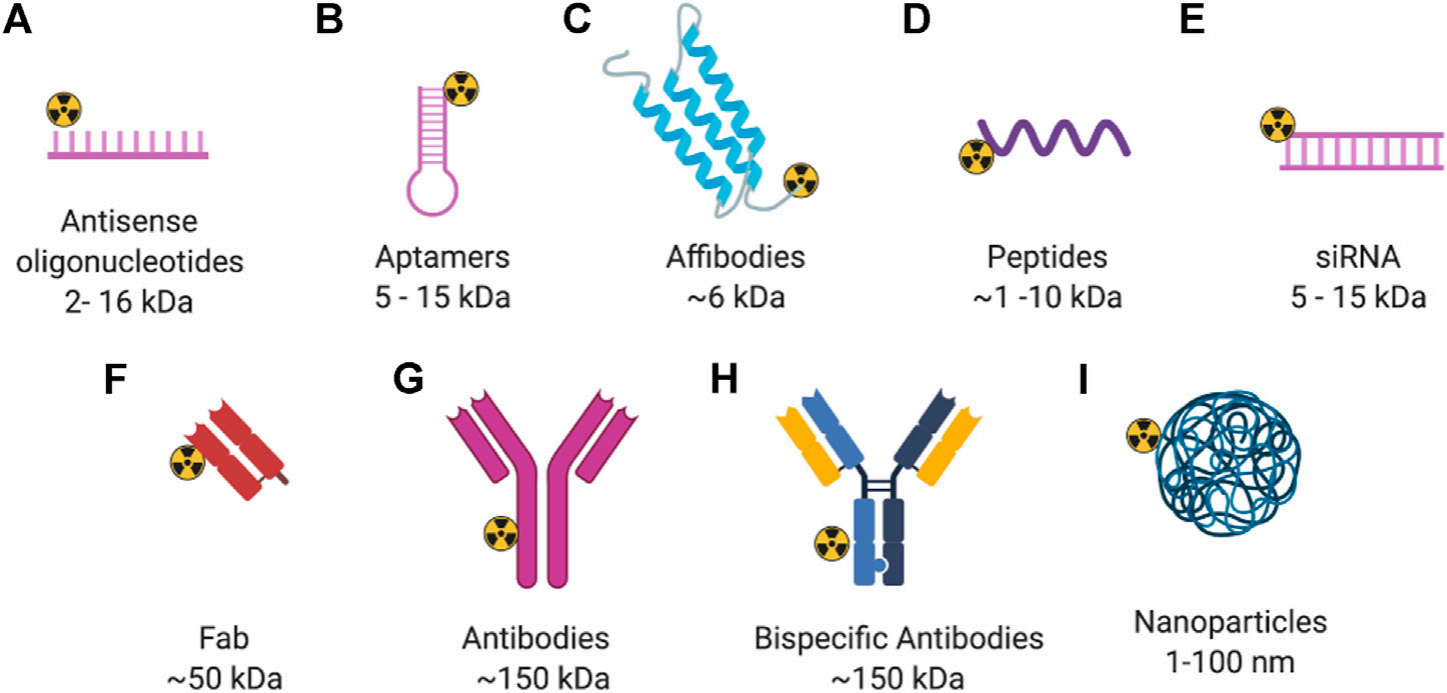
General structure of radiolabeled biomolecules for imaging of breast cancer. Created with BioRender.com.

Currently, the diagnosis of breast cancer still depends on biopsy followed by tissue analysis using immunohistochemistry (IHC) or fluorescence *in situ* hybridization (FISH) to define receptor status and guide treatments. However, tissue analysis results may not be accurate due to several limitations, such as sampling errors, tumor heterogeneity, and changes in receptor status over the course of treatment (de Vries et al., 2019). Thus, non-invasive imaging of therapeutic targets or distribution of therapeutic agents throughout the whole body via PET or SPECT with radiolabeled agents can serve as a complementary diagnostic technique to tissue-based analyses. The type of imaging agent for nuclear imaging is also diverse and includes small molecules, peptides, antibodies, affibodies, antisense oligonucleotides, bispecific scaffolds, and nanoparticles. Figures 1, 2 show the diverse set of probes described in this review.

Herein, we highlight recent advances in preclinical imaging and targeted therapy, the combination of which form the basis of theranostics, for the different molecular subtypes of breast cancer. Table 1 lists the different imaging agents described in this review. We also draw attention to the different cell lines tested in preclinical studies, which represent each molecular subtype of breast cancer to help guide future investigations of novel theranostic agents.

**TABLE 1:** Summary of the preclinical imaging agents discussed in this review for the different subtypes of breast cancer.

Target	Agent	Type of structure	Tumor model	Imaging modality	References
Luminal-subtype					
ER	^99m^Tc-DTPA-estradiol	Small molecule	MCF-7	SPECT	Xia et al. (2016)
	^99m^Tc(V)-nitrido complex **C**	Small molecule	MCF-7	None	Tejería et al. (2019)
CXCR4	^99m^Tc-AMD3465	Small molecule	MCF-7	SPECT/CT	Zhang et al. (2018)
GRPR	^111^In-JMV4168	Peptide	T47D	SPECT/CT	Dalm et al. (2015)
CDK4/6	^18^F-CDKi	Small molecule	MCF-7	PET/CT	Ramos et al. (2020)
	^99m^Tc-labeled palbociclib analogs	Small molecule	MCF-7	SPECT/CT	Song et al. (2019)
	^99m^Tc-tricine-TPPTS-L	Small molecule	MCF-7	SPECT/CT	Gan et al. (2020)
PI3K	^11^C-pictilisib (GDC-0941)	Small molecule	MCF-7	PET	Han et al. (2019)
	^18^F-PEG_3_-GDC-0941	Small molecule	MCF-7	PET	Altine et al. (2019)
Sigma-1 receptor	^124^I-IPAG	Small molecule	MCF-7	PET	Gangangari et al. (2020)
MDM2	^99m^Tc-HYNIC-ASON	Antisense oligonucleotide	MCF-7	SPECT	Fu et al. (2010)
**HER2-subtype**					
HER2	^18^F-aptamer	Aptamer	BT-474	PET	Kim et al. (2019)
HER3	^111^In-HEHEHE-Z08698-NOTA	Affibody	BT-474	SPECT	Andersson et al. (2015)
GRPR	^68^Ga-NOTA-PEG_3_-RM26	Peptide	BT-474	PET/CT	Varasteh et al. (2014)
VPAC	^68^Ga-NODAGA-peptide	Peptide	BT-474	PET/CT	Kumar et al. (2019)
Cobalamin (cbl)	^89^Zr-cbl	Small molecule	MDA-MB-453	PET	Kuda-Wedagedara et al. (2017)
**Triple-negative breast cancer**					
EGFR	^99m^Tc-PmFab-His6	Fab	MDA-MB-468	SPECT/CT	Ku et al. (2019a)
CMKLR1	^68^Ga-DOTA-ADX-CG34	Peptide	DU4475	PET/MR	Erdmann et al. (2019)
HDAC	^64^Cu-HDACi	Small molecule	MDA-MB-231	PET/CT	Meng et al. (2013)
MYC	^89^Zr-transferrin	Protein	MDA-MB-231; MDA-MB-157	PET	Henry et al. (2018)
TF	^64^Cu-NOTA-ALT-836-fab	Fab	MDA-MB-231	PET	Shi et al. (2015)
CXCR4	^99m^Tc-HYNIC-siRNA1	siRNA	MDA-MB-231	SPECT	Fu et al. (2016)
MUC1	^99m^Tc-S1-apMUC1	Nanoparticle-aptamer conjugate	MDA-MB-231	SPECT	Pascual et al. (2017)
**Dual-receptor**					
GRPR/FA	^99m^Tc-BBN-FA	Bispecific peptide	T47D	SPECT/CT	Aranda-Lara et al. (2016a)
	^177^Lu-BBN-FA	Bispecific peptide	T47D	SPECT/CT	Aranda-lara et al. (2016b)
GRPR/ NPY(Y_1_)R	^68^Ga-24	Bispecific peptide	T47D	PET/CT	Vall-Sagarra et al. (2018)
α_v_β_3_/CD13	^68^Ga-NGR-RGD	Bispecific peptide	MCF-7	PET/CT	Gai et al. (2020)
EGFR/HER2	^64^Cu-NOTA-fab-PEG24-EGF	Bispecific fab	MDA-MB-231-H2N	PET/CT	Kwon et al. (2017)
T-cell/CEA	^89^Zr-AMG211	Bispecific antibody	BT-474	PET	Waaijer et al. (2018)
EGFR/c-MET	[^89^Zr]ZrDFO-amivantamab	Bispecific antibody	MDA-MB-468; MDA-MB-231; MDA-MB-453	PET/CT	Cavaliere et al. (2020)

## Preclinical Imaging Agents for the Different Subtypes of Breast Cancer

### Luminal Subtype

The luminal subtype of breast cancer accounts for about 70% of all breast cancer patients and encompasses molecular signatures that are ER and/or PR positive, and HER2 negative (Waks and Winer, 2019). As the luminal subtype is the most common subtype of breast cancer, many research efforts have been focused on cell surface receptors and intracellular targets for development of both molecular imaging and targeted therapy of luminal breast cancer. Summarized below are some of the most promising agents for PET and SPECT imaging of the luminal subtype of breast cancer.

#### Cell Surface Receptor

Approximately 70% of breast cancers express ER or PR, or both. Hence, endocrine therapy is the most important treatment for the luminal subtype of breast cancer. Imaging of ER and PR can help determine the status of the tumor tissue, and thus predict prognosis and efficacy of endocrine therapy. A recent review highlighted the preclinical and clinical research progress within the last 5 years of ER imaging with ^18^F-fluoroestradiol (^18^F-FES) and PR imaging with ^18^F-fluorofuranyl norprogesterone (^18^F-FFNP) in breast cancer (Kumar et al., 2020). Here, we summarized some promising studies that showed imaging of other targets in breast cancer luminal subtype xenografts.

Typically, the MCF-7 and T47D xenograft models are used as high ER-expressing animal models while the MDA-MB-231 xenograft is used as a low ER-expressing model in many of the studies that we summarize herein (Dai et al., 2017). One SPECT imaging agent for ER is an estradiol analog labeled with ^99m^Tc (t_1/2_ = 6 h) using diethylenetriamine pentaacetate (DTPA) as the chelate to afford ^99m^Tc-DTPA-estradiol (Xia et al., 2016). High tumor uptake was found in MCF-7 xenografts with 6.1 ± 0.38 %ID/g at 4 h post-injection (p.i.), and high tumor-to-blood (T/B) ratio of 2.8 ± 0.39. As expected, low tumor uptake was observed in the MDA-MB-231 xenografts. Nonetheless, high uptake that amounted to 50% ID/g at 4 h p. i. in the liver, which is a common site for metastasis of breast tumor (Ma et al., 2015), may limit the detection of metastatic lesions in this organ when ^99m^Tc-DTPA-estradiol is translated to clinical studies. Another derivative of estradiol, ^99m^Tc(V)-nitrido complex C (^99m^Tc-C, Figure 1), was also developed for SPECT imaging of ER (Tejería et al., 2019). Uptake of ^99m^Tc-C in the liver, at 1.1 ± 0.38% ID/g at 2 h p. i., was much lower than that of ^99m^Tc-DTPA-estradiol. However, its tumor uptake was also very low (0.59 ± 0.12% ID/g), which resulted in a low T/B ratio of only 0.35 ± 0.19 at 1 h p. i., i.e., lower concentration in tumor tissue than blood. Differences in tumor uptake of these two estradiol-derived probes might be due to their different chemical structures that can affect pharmacokinetic properties and receptor-binding specificity. Hence, further optimization is still needed to improve ER targeting and pharmacokinetic properties and provide better contrast between metastatic lesions and surrounding normal tissue.

The chemokine receptor 4 (CXCR4), a seven-transmembrane G protein-coupled receptor (GPCR), is another promising target for theranostic development (Kircher et al., 2018). CXCR4 is expressed in 67% of breast cancer cells, with a level double of that in normal breast tissues (Salvucci et al., 2006). Several studies demonstrated that CXCR4 expression may have value in predicting breast cancer prognosis (Xu et al., 2015). For examples, breast cancer patients with high levels of CXCR4 were found to have more extensive metastasis to lymph nodes (Kato et al., 2003) and significantly reduced disease-free survival and overall survival (Zhang et al., 2014a). There are several CXCR4 inhibitors under clinical trials for the treatment of multiple myeloma, small cell lung cancer, and leukemia (Cooper et al., 2017; Salgia et al., 2017; Ghobrial et al., 2019), although none has been reported for breast cancer. AMD3465, a small molecule antagonist of CXCR4, was labeled with ^99m^Tc to obtain ^99m^Tc-AMD3465 (Figure 1) (Zhang et al., 2018). It showed a moderate tumor uptake of 2.1 ± 0.39% ID/g, but high T/B ratio of 9.4 at 1 h p. i. Specific binding of ^99m^Tc-AMD3465 to CXCR4 was demonstrated by treatment with excess unlabeled AMD3465 which reduced tumor uptake by 36%.

Another example of a cell surface receptor is the gastrin-releasing peptide receptor (GRPR), which is a G-protein coupled receptor highly expressed in the pancreas and lowly expressed in the normal breast tissues (Baratto et al., 2020). In autoradiography studies, GRPR was reported to be expressed at high density (>2000 dpm/mg tissue) in 74% (50 of 68) of breast cancer tissues from patients (Reubi et al., 2002).

**FIGURE 3:**
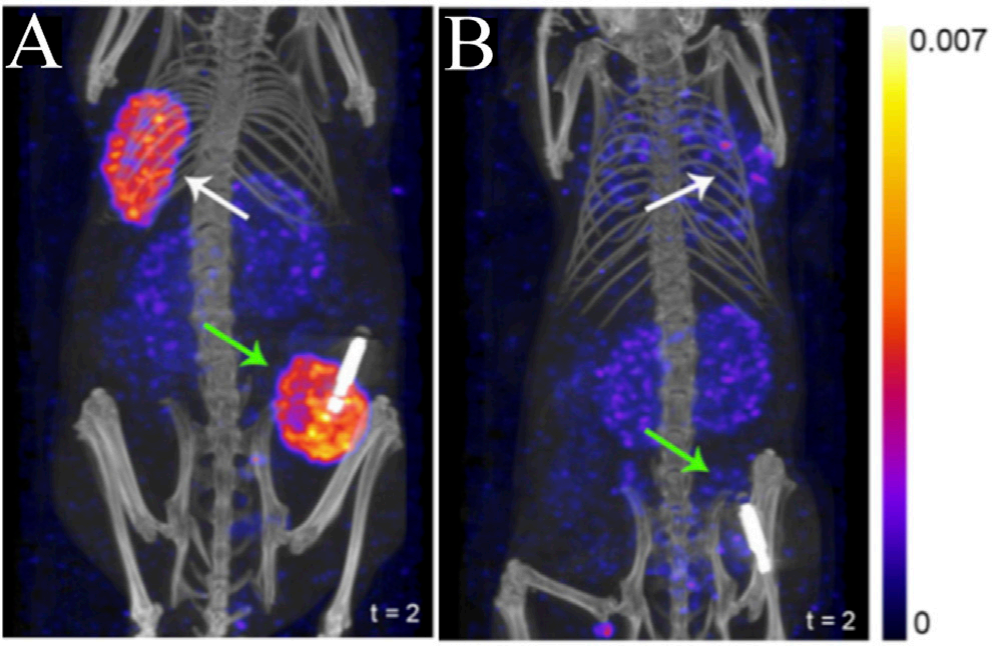
SPECT/CT imaging using ^111^In-JMV4168 in orthotopic (green arrows) and subcutaneous (white arrows) tumors of T47D **(A)** and MCF7 **(B)** xenografts at 4 h post-injection of ^111^In-JMV4168 (Dalm et al., 2015). The general structure of ^111^In-JMV4168 was shown in Figure 2D.

Evaluating radiotracers that bind to GRPR typically employ the T47D xenograft model, as this cell line express high levels of GRPR. Agonists and antagonists of GRPR have been adapted as SPECT imaging agents. ^111^In-AMBA (^111^In t_1/2_ = 2.8 days) is a GRPR agonist that has been tested in an autoradiography study of 50 human breast cancer tissue specimens, with 96% (48/50) of them showing elevated GRPR levels (Dalm et al., 2015). A positive correlation was also found between ER status and GRPR expression. Those results were in agreement with previous studies, where a positive correlation between ER expression and GRPR binding affinity was detected in human breast cancer samples (Halmos et al., 1995). ^111^In-JMV4168 (Peptides, Figure 2), a GRPR antagonist, was developed for SPECT/CT imaging (Dalm et al., 2015). Tumor uptake was about 5% ID/g in both subcutaneous and orthotopic tumors of T47D xenografts (Figure 3). Autoradiography showed high binding of ^111^In-JMV4168 in T47D xenografts. In orthotopic tumor tissues, ^111^In-JMV4168 bound with 2.2 ± 4% of added dose (%AD), whereas low binding was observed in the blocking group with 0.1% AD, confirming the specific binding of ^111^In-JMV4168 to GRPR *ex vivo* (Dalm et al., 2015).

#### Intracellular Targets

Cell cycle dysregulation leads to uncontrolled progression to tumor (Schafer, 1998). The cell cycle consists of 4 phases: DNA replication (S), mitosis (M), and two gaps (G1 and G2) between the S and M phases. Cyclin-dependent kinases 4/6 (CDK4/6) are two kinases that control cell cycle from G1 to S phase (Lee et al., 2019). CDK4/6 overexpression occurs in many cancers, including breast cancer (O’Sullivan et al., 2019; VanArsdale et al., 2015). Palbociclib, a CDK4/6 inhibitor (CDKi), is in phase 3 clinical trials for the treatment of ER-positive advanced breast cancer (Finn et al., 2016). The use of radiolabeled CDKi for tumor imaging has gained increased attention as companion diagnostic imaging agents to these inhibitors. The palbociclib CDKi was labeled with ^18^F (t_1/2_ = 110 min) to obtain ^18^F-CDKi (Figure 1), for PET imaging of MCF-7 xenografts (Ramos et al., 2020). Tumor uptake was high at about 4% ID/g at 2 h p. i for ^18^F-CDKi, which translated into a high T/B ratio of about 5. The tumor uptake decreased to 0.3% ID/g at 2 h p. i. when mice were blocked with excess of palbociclib.

A series of ^99m^Tc-labeled palbociclib analogs were also developed for SPECT imaging (Song et al., 2019). However, tumor uptake was moderate, and T/B ratios were low for the ligand series, accompanied by very high liver uptake of more than 50% ID/g. In this study, ^99m^Tc-L2 showed the highest tumor uptake of 2.7 ± 0.26% ID/g but low T/B ratio of 0.42 at 2 h p. i. However, the highest radiotracer accumulation was observed in the liver of greater than 50% ID/g from ^99m^Tc-L2 to L5, which might be due to their relatively high lipophilicity (Log *P* = 1.5). To reduce the liver uptake, the chelator was changed from an isocyano-group to an hydrazinonicotinamide (HYNIC) moiety, where tricine/TPPTS were used as co-ligands to afford a new ^99m^Tc-labeled palbociclib complex, ^99m^Tc-tricine-TPPTS-L (Figure 1) (Gan et al., 2020). The Log *P* of the ^99m^Tc-tricine-TPPTS-L variant was −2.9 ± 0.1, which was much lower than that of ^99m^Tc-L2 to L5, demonstrating that ^99m^Tc-tricine-TPPTS**-**L was more hydrophilic. Tumor uptake of ^99m^Tc-tricine-TPPTS-L was good, at 3.8 ± 1.3 and 2.7 ± 0.58% ID/g at 1 and 2 h p. i., respectively, albeit with low T/B ratio of about 0.4. The liver uptake was indeed much reduced to as low as 4.2 ± 0.33% ID/g at 2 h p. i. These studies showed that radiolabeled palbociclib analogs may have the potential to image CDK4/6 via PET or SPECT as a companion diagnostic agent to CDK4/6 inhibitors.

Another intracellular kinase that also regulates cell proliferation, survival, and migration is the phosphatidylinositol 3-kinase (PI3K) (Bader et al., 2005). Abnormal activation of PI3K/Akt/mTOR has been found in about 70% of breast cancer cases (Cancer-Genome-Atlas-Network, 2012). Pictilisib (GDC-0941), a PI3K inhibitor, is currently under phase Ib clinical trial in patients with advanced breast cancer and non-small cell lung cancer (Yamamoto et al., 2017; Schöffski et al., 2018). Pictilisib was labeled with ^11^C (t_1/2_ = 20 min) for PET imaging in pictilisib-sensitive MCF-7 xenograft models (Han et al., 2019). The tumor uptake of ^11^C-pictilisib was 2.9 ± 0.07% ID/g with T/B ratio of 2.1 ± 0.34 at 1 h p. i. in these xenograft models, demonstrating excellent tumor penetration regardless of the short half-life of ^11^C. In contrast, PET imaging with ^11^C-pictilisib in pictilisib-resistant MDA-MB-231 xenograft models showed significantly decreased tumor uptake. However, uptake in the liver was the highest. To reduce liver uptake, a triethylene glycol di (*p*-toluenesulfonate) (TsO-PEG_3_-OTs) modified agent, ^18^F-PEG_3_-GDC-0941, was developed (Altine et al., 2019). Its liver uptake was 4.7 ± 0.86 %ID/g at 1 h p. i., which is about 76% lower than that of ^11^C-pictilisib. ^18^F-PEG_3_-GDC-0941 also showed high and specific tumor uptake, indicating that imaging PI3K could be a potential strategy for monitoring response to pictilisib treatment.

Sigma-1 receptors (S1R) is a unique ligand-regulated membrane protein involved in modulating cellular protein and lipid homeostasis (Maher et al., 2018). S1R mRNA was found to be overexpressed in 64% of breast cancer tissues, and in several ER-positive breast cancer cell lines on the cell membrane and in the endoplasmic reticulum (Wang et al., 2004). The small molecule inhibitor of S1R 1-(4-Iodophenyl)-3-(2-adamantyl)guanidine (IPAG) was shown to decrease the expression of the programmed death receptor ligand 1 (PD-L1) and suppress PD-L1 interaction with its PD-1 receptor in T-cell, and in cell lines of PC3 prostate cancer and MDA-MB-231 triple negative breast cancer (Maher et al., 2018). IPAG was labeled with ^124^I (t_1/2_ = 4.18 days) (Figure 1) for PET imaging in MCF-7 xenografts (Gangangari et al., 2020). The tumor uptake of ^124^I-IPAG was 1.1 ± 0.24 and 0.94 ± 0.22% ID/g at 24 and 48 h p. i., respectively, with extremely high T/B ratio of 22 ± 6.6 and 46 ± 10.0. With the specific targeting and the high T/B ratios, ^124^I-IPAG holds great potential for imaging S1R in tumor and may be used to help to define the interaction between S1R and PD-L1 as a consequence of S1R-targeted or checkpoint inhibitor therapy.

**FIGURE 4:**
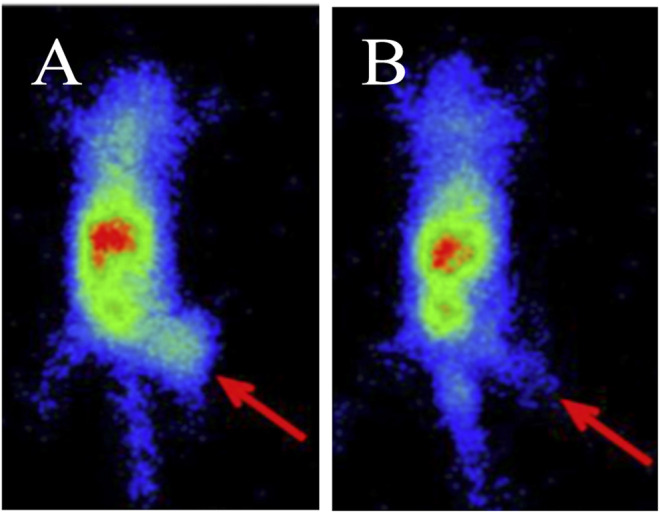
SPECT Imaging of MDM2 expression in MCF-7 xenografts using ^99m^Tc-HYNIC-antisense **(A)** and mismatch **(B)** probes at 4 h post-injection. Tumors are indicated by red arrows (Fu et al., 2010). The general structure of ^99m^Tc-HYNIC-antisense was shown in Figure 2A.

Apart from protein targets, oncogenes are also attractive targets in breast cancer. Mouse double-minute 2 (MDM2), an oncogene, is regarded as the major negative regulator of the function of the p53 tumor suppressor, and found to be overexpressed in many malignant tumors, including breast cancer (Haupt et al., 2017). High expression of MDM2 with the consequent inactivating of p53 is associated with tumor development (Graat et al., 2007, Zhang et al., 2017). The ^99m^Tc-labeled antisense oligonucleotides (ASONs) (Antisense oligonucleotides, Figure 2) has been used to visualize MDM2 mRNA expression in MCF-7 xenografts through SPECT imaging (Figure 4) (Fu et al., 2010). This antisense probe and its mismatched oligonucleotide control have similar biodistribution properties in normal organs with fast blood clearance. The tumor uptake of the probe was high and steady from 9.2 ± 1.4 to 8.1 ± 1.1% ID/g at 1 and 6 h p. i., respectively, with increasing T/B ratio of 1.24 at 1 h p. i. to 4.11 at 6 h p. i.. In contrast, tumor uptake of the mismatched oligonucleotide control was significantly lower. This study demonstrates the feasibility of specifically targeting MDM2 mRNA with ^99m^Tc-HYNIC-ASON. With increasing evidence showing that antisense oligonucleotides contribute to breast cancer treatment (Yang et al., 2003), *in vivo* imaging with radiolabeled antisense oligonucleotides may provide a tool to monitor therapeutic response.

### HER2 Subtype

The HER2 subtype of breast cancer is classified by amplification of the HER2 oncogene and overexpression of the HER2 transmembrane receptor tyrosine kinase (Ross et al., 2009). HER2 belongs to the epidermal growth factor receptor family, consisting of HER1/EGFR, HER3, and HER4 (Wang, 2017). HER2 is amplified in 15–20% of all breast cancers, and the HER2 subtype is associated with more aggressive growth and poor prognosis (Waks and Winer, 2019). One of the previous reviews has presented a comprehensive summary of recent advances in HER2-targeted imaging and therapy in nuclear medicine prior to 2018 (Massicano et al., 2018). Herein, we summarize new findings in preclinical studies from 2018 to 2020. We will also discuss imaging studies with other novel targets that evaluate xenograft models within the HER2 subtype. In these recent studies, the BT-474 xenograft model is typically used due to its overexpression of HER2, high tumorigenicity in standard immune compromised mice strains, and high sensitivity to HER2-targeted treatments.

#### HER2

Aptamers are single-stranded oligonucleotides that have unique three-dimensional shape to specifically and tightly bind to their protein targets (Ireson and Kelland, 2006). Currently, many therapeutic aptamers are under clinical investigation, including a nucleolin-targeted DNA aptamer for the treatment of renal cell carcinoma (Rosenberg et al., 2014) and several anti-VEGF aptamers for macular degeneration and angioma (Eyetech-Study-Group, 2002; Dahr et al., 2007). One recent study demonstrated that a HER2-targeted aptamer can differentiate both HER2-positive breast cancer cells and xenografted mice models from other subtypes of breast cancer through fluorescence imaging (Liu et al., 2018). SH-1194-35, a HER2-targeted DNA aptamer, was labeled with ^18^F using click chemistry between the amine-terminal and an *N*-succinimidyl 4-^18^F-fluorobenzoate (^18^F-SFB) to form an amide linkage (Aptamer, Figure 2) (Kim et al., 2019). Tumor uptake of this ^18^F-labeled HER2 aptamer in BT474 xenograft models was rather low at 0.62 ± 0.04% ID/g at 1 h p. i., but was higher than that in the HER2-negative MDA-MB-231 xenograft models. The highest uptake was observed in the intestines and kidneys. Although optimization is still needed to increase uptake in the tumor and reduce uptake in normal organs, the translational potential of aptamers to clinical studies opens a new direction for HER2-targeted therapy against breast cancer.

#### HER3

HER3 overexpression is a resistance mechanism to several anticancer therapies, including hormone therapy in breast cancer (Johnston et al., 2016). Imaging HER3 may help to explain the mechanism of resistance to standard of care treatments. This approach could also predict the HER3-targeted therapeutic efficacy of tyrosine kinase inhibitors neratinib and anti-HER3 antibody patritumab in advanced solid tumors, including breast cancer (Sergina et al., 2007; LoRusso et al., 2013; Mukai et al., 2016; Hyman et al., 2018). Andersson et al. developed a radiolabeled anti-HER3 affibody ^111^In-HEHEHE-Z08698-NOTA (Affibody, Figure 2) which had extremely high binding affinity for HER3 (K_D_ of 5.4 ± 0.4 pM) (Andersson et al., 2015). The tumor uptake of ^111^In-HEHEHE-Z08698-NOTA in the BT-474 xenograft models was 5.1 ± 0.4 and 3.7 ± 0.2 at 1 and 24 h p. i., respectively, and T/B ratio increased from 5.3 ± 0.4 to 15.5 ± 0.7 at these time points. These properties are desirable for clinical translation, given the feasibility of same-day imaging of HER3 expression.

#### Other Targets in HER2-Positive Models

##### GRPR

GRPR is also expressed in HER2-positive cells. GRPR belongs to the mammalian bombesin (BBN)-like peptide receptor family (Qu et al., 2018). BBN is a 14-amino acid peptide, originally found in the frog skin (Erspamer et al., 1970; Jensen et al., 2008). One of its mammalian homologs is a gastrin-releasing peptide (GRP) (Qu et al., 2018), which binds specifically to GRPR. RM26 (D-Phe-Gln-Trp-Ala-Val-Gly-His-Sta-Leu-NH2), an antagonist analog of BBN, was conjugated to different lengths of polyethylene glycol (PEG_x_) and the NOTA chelator for radiolabeling with ^68^Ga to obtain ^68^Ga-NOTA-PEG_x_-RM26 (Peptides, Figure 2) to optimize the radiotracer’s targeting efficiency (Varasteh et al., 2014). ^68^Ga-NOTA-PEG_3_-RM26, with a three PEG unit linker, was found to have the lowest liver uptake of 0.7 ± 0.1% ID/g at 2 h p. i. Tumor uptake in the BT-474 xenograft models was 2.8 ± 0.4% ID/g with an extremely high T/B ratio of 42 ± 5 at 2 h p. i. Therefore, it appears that biological properties can be optimized by insertion of an appropriate length of the PEG spacer between the peptide and radiometal chelator. Since PEG has been widely used for modification of therapeutic peptides and proteins to reduce enzymatic degradation (Roberts et al., 2002), the above study presents a rational approach for optimizing the *in vivo* pharmacokinetic and binding properties of peptide probes.

##### VPAC

TP-3805 is an analog of the pituitary adenylate cyclase-activating peptide (PACAP), which has high affinity for the VPAC [combination of vasoactive intestinal peptide (VIP) and PACAP] receptors (Thakur et al., 2013). These receptors are highly expressed in malignant breast cancer. TP-3805 was conjugated to either 1,4,7-triazacyclononane,1-glutaric acid-4,7-acetic acid (NODAGA) or 1,4,7,10-tetraazacyclododecane-1,4,7,10-tetraacetic acid (DOTA) chelate for radiolabeling with ^68^Ga to obtain ^68^Ga-NODAGA-peptide or ^68^Ga-DOTA-peptide, respectively (Peptides, Figure 2). PET imaging with these radiotracers was performed to compare their *in vivo* stability and pharmacokinetic properties in breast cancer xenografts (Kumar et al., 2019). Tumor uptake of ^68^Ga-NODAGA-peptide in the BT-474 xenograft models was 2.4 ± 0.3% ID/g at 1 h p. i., with tumor to muscle (T/M) ratio of 3.4 ± 0.3 whereas ^68^Ga-DOTA-peptide showed similar tumor uptake but a lower T/M ratio of 1.9 ± 0.9 at 1 h p. i. Further, ^68^Ga-NODAGA-peptide also showed more flexibility in radiolabeling, higher stability *in vitro*, and higher cell binding affinity than ^68^Ga-DOTA-peptide. These differences may be due to differences in coordination chemistry. For example, Ga(III) uses all 11 of its coordination sites to form a complex with NODAGA, whereas two sites remain uncoordinated when DOTA is used as a chelator (Viola-Villegas and Doyle, 2009). Although the *in vivo* stability of both ^68^Ga-NODAGA- and ^68^Ga-DOTA-labeled TP-3805 needs improvement, this study suggests that changing the chelator is a strategy to optimize the pharmacologic properties of probes.

##### Cobalamin

Vitamin B_12_, or cobalamin (Cbl), is an essential nutrient required to maintain cell growth and differentiation (Gherasim et al., 2013). Cbl is transported by binding to the transport protein transcobalamin, which is recognized by specific receptors such as CD320 (Quadros and Sequeira, 2013), which is highly expressed in several cancers, including breast cancer (Sysel et al., 2013). Cbl was radiolabeled with ^89^Zr (t_1/2_ = 78.4 h) and used for PET imaging (Kuda-Wedagedara et al., 2017). Tumor uptake of ^89^Zr-Cbl (Figure 1) in HER2-positive MDA-MB-453 xenograft models was 3.8 ± 0.77% ID/g with T/B ratio of about 9.7 at 48 h p. i. In addition, clearance from blood was evident from 4 to 48 h with approximately 90% decrease in activity concentration by 48 h p. i. A drawback of this radiotracer is its high uptake in the kidney. This study demonstrates the feasibility of labeling vitamin B12 as a tracer and use it for breast cancer imaging.

In summary, HER2 remains an important target for theranostic development. Several other promising targets in the HER2 subtype of breast cancer such as HER3, GRPR, and vitamin B12 offer additional options for targeted therapy, with their respective companion diagnostic imaging agents readily available for assessing target engagement or monitoring response to treatment. Further investigations are still needed for optimization and validation of these nuclear imaging agents.

### Triple-Negative Breast Cancer

Triple-negative breast cancer (TNBC) is characterized by the absence of ER and PR expression, or lack of HER2 overexpression (Waks and Winer, 2019). TNBC makes up approximately 15% of all breast cancers (Waks and Winer, 2019). The absence of these receptors has long limited the treatment of patients with TNBC to chemotherapy, with its accompanying serious adverse effects and drug resistance. Hence, patients with TNBC are faced with a grim prospect of poor prognosis, high rate of distant metastasis and short survival time (Bianchini et al., 2016; He et al., 2018). However, a new era in TNBC treatment has recently begun with the FDA-approval of drugs targeting PD-L1 (e.g., atezolizumab), and trophoblast antigen 2 (Trop-2) (e.g., sacituzumab govitecan) (Bardia et al., 2019; Cyprian et al., 2019). Development of new PET and SPECT imaging agents that inform on the status of new therapeutic targets could help guide treatment options for patients with TNBC. In the preclinical studies of new imaging probes described below, MDA-MB-231 and MDA-MB-468 xenograft models are typically used as animal models for TNBC.

#### EGFR

TNBC patients with higher expression of the epidermal growth factor receptor (EGFR) have shorter overall survival (Vallböhmer et al., 2005, Zhang et al., 2003). Panitumumab combined with chemotherapy showed promising results in a phase II clinical trial (Cowherd et al., 2015). Thus, imaging EGFR with a radiolabeled panitumumab Fab (PmFab) could afford a tool to monitor response to this combination treatment. The SPECT imaging agent ^99m^Tc-PmFab-His_6_ (Fab, Figure 2) was prepared by conjugating PmFab and the hexahistidine peptide (His_6_) which serves as a chelate for ^99m^Tc labeling (Ku et al., 2019a). Tumor uptake in MDA-MB-468 xenograft models was 15 ± 3.1% ID/g with T/B ratio of 12 ± 1.4 at 24 h p. i., indicating that ^99m^Tc-PmFab-His_6_ is a promising probe for imaging EGFR and may be used to monitor the response to EGFR-directed therapies.

#### Chemokine-like Receptor 1

Chemerin is known to be involved in angiogenesis, cancer-related inflammation, and insulin resistance (Perumalsamy et al., 2017). The chemokine-like receptor 1 (CMKLR1) is a chemotactic cellular receptor for chemerin (Pachynski et al., 2019). CMKLR1 and chemerin have recently been recognized as modulators of tumor proliferation (Shin and Pachynski, 2018). Increasing chemerin expression in the breast tumor microenvironment can suppress tumor growth (Pachynski et al., 2019). Further, high mRNA expression of CMKLR1 is associated with a longer relapse-free survival of breast cancer patients (Treeck et al., 2019). The first imaging of CMKLR1 *in vivo* was performed with a family of five novel CMKLR1 peptides derived from chemerin-9 and labeled with ^68^Ga (Erdmann et al., 2019) (Peptides, Figure 2). One of the radiotracers, ^68^Ga-DOTA-ADX-CG34, showed the highest tumor uptake with 6.2 ± 0.5% ID/g in CMKLR1-positive DU4475 (TNBC) xenograft models, while ^68^Ga-DOTA-AHX-CG34 presented the highest T/B ratio of 5.9 ± 0.7 at 1 h p. i., and ^68^Ga-DOTA-KCap-CG34 the lowest kidney and liver uptake. Since high CMKLR1 expression is associated with longer relapse-free survival, CMKLR1-targeted probes are promising prognostic tools for breast cancer.

#### Histone Deacetylases

Histone deacetylases (HDACs) are a class of enzymes that modulate transcription and therefore alter gene expression (Bolden et al., 2006; Falkenberg and Johnstone, 2014). Four HDAC inhibitors (HDACi), namely romidepsin, panobinostat, vorinostat, and belinostat, have been approved by the Food and Drug Administration (FDA) for the treatment of T-cell lymphoma and multiple myelomas, while several other HDACi compounds are under clinical investigation (Singh et al., 2018). Therefore, imaging HDAC is needed for non-invasive assessment of its expression in the body and prediction of response to HDAC-targeted treatment. Preclinical studies have shown that HDACi is toxic to TNBC cells and decreases tumorigenesis *in vivo* (Tate et al., 2012). CUDC-101, a small molecule HDACi, is currently in phase I clinical trials for the treatment of advanced breast cancer (Shimizu et al., 2014). CUDC-101 was labeled with ^64^Cu to obtain ^64^Cu-HDACi (Figure 1) for PET imaging of TNBC xenografts (Meng et al., 2013). Tumor uptake of ^64^Cu-HDACi in was 2.2 ± 0.18% ID/g, as well as high T/B ratio of 4.4 ± 0.88 at 24 h p. i. with moderate uptake in the liver and kidney (3.2 ± 1.3 and 1.9 ± 0.06% ID/g, respectively). Thus, ^64^Cu-HDACi shows promise for clinical translation to monitor the response to HDACi treatment in breast cancer.

#### C-Myc Proto-Oncogene

The c-myc proto-oncogene (MYC) is known to play important roles in mRNA regulation, cell proliferation, cell metabolism, and cell death (Horiuchi et al., 2012; Stine et al., 2015). MYC expression is found in 87% of TNBC patients (164 of 187) and associated with poor survival (Bouchalova et al., 2015). There is also evidence that MYC overexpression contributes to drug resistance in patients with TNBC (Carey et al., 2018, Lee et al., 2017). However, directly targeting of the MYC gene remains a challenge, and alternate approaches have been developed (Horiuchi et al., 2014). It has been shown that upregulation of MYC leads to increased surface expression of transferrin receptor (TfR) (O’Donnell et al., 2006), hence ^89^Zr-transferrin was developed as a potential probe for MYC status and tumor burden in several cancer models, such as prostate cancer and lymphoma (Holland et al., 2012; Doran et al., 2016). Recently, PET imaging with ^89^Zr-transferrin in TNBC models has also been performed using MDA-MB-231 and MDA-MB-157 xenografts, showing similar accumulation of the radiotracer in both models with 4% ID/g at 48 h p. i. (Henry et al., 2018). In patient-derived xenograft models of TNBC PET imaging with ^89^Zr-transferrin at 48 h p. i. delineated xenografted tumors from normal organs, indicating the potential of ^89^Zr-transferrin as a probe for MYC to monitor response to treatments that modulate this oncogene.

#### Tissue Factor

Tissue factor (TF), also known as thrombokinase or CD142, has been confirmed to be overexpressed on TNBC cells (Callander et al., 1992). Importantly, a high level of TF also contributes to progression and poor survival in TNBC patients (Ruf et al., 2010; Ueno et al., 2000). ALT-836, a chimeric anti-human TF monoclonal antibody (mAb), has been used for the treatment of solid tumors that overexpress TF in a clinical trial (Clinical Trials.gov Identifier: NCT01325558). A radiolabeled antibody fragment, ^64^Cu-NOTA-ALT-836-Fab (Fab, Figure 2), was developed and shown to have an uptake level of about 4% ID/g with T/B ratio of 2 at 24 h p. i. in MDA-MB-231 xenograft models (Shi et al., 2015). Therefore, targeting TF could be a potential way for imaging and therapy of TNBC.

#### CXCR4

Similar to its involvement in the luminal subtype, CXCR4 is also a potential target for theranostics of TNBC using RNA interference (RNAi) technology, a powerful tool in gene therapy research (Bottai et al., 2017). The delivery of small-interference RNA (siRNA) can affect the efficacy of RNAi therapy *in vivo* (Chen et al., 2018). A^99m^Tc-labeled siRNA was used to target CXCR4 in breast cancer xenografts for tracing the delivery of siRNAs *in vivo* (Fu et al., 2016). Due to its fast blood clearance, the tumor uptake of ^99m^Tc-HYNIC-siRNA1 (siRNA, Figure 2) in MDA-MB-231 xenograft models increased from 4.5 ± 0.47 to 8.4 ± 1.1% ID/g at 1 and 6 h p. i., respectively, with corresponding increase in T/B ratio from 0.6 to 4.8. In comparison, tumor uptake of the siRNA control was lower, indicating specific targeting of ^99m^Tc-HYNIC-siRNA1 to breast cancer. This probe may be a useful tool to predict the efficacy of RNAi gene therapy.

#### Mucin 1

Mucin 1 (MUC1) is a cell surface glycoprotein and expressed in over 90% of all breast cancers (Miller-Kleinhenz et al., 2015) and 94% of the TNBC subtype (Siroy et al., 2013). High expression of MUC1 has also been found to be associated with metastases and poor survival (Kim et al., 2020, McGuckin et al., 1995), and MUC1 has been reported to contribute to immune escape in TNBC, indicating that MUC1 is a potential immunotherapeutic target for TNBC (Maeda et al., 2018). Several clinical trials targeting MUC1 are ongoing in breast cancer patients (Apostolopoulos et al., 2006; Ibrahim et al., 2011, Tang et al., 2017). Therefore, MUC1 is recognized as a promising marker for theranostics of breast cancer and has been targeted for imaging agent development. One example is ^99m^Tc-labeled mesoporous silica nanoparticles (MSNs), ^99m^Tc-S1-apMUC1 (Nanoparticles, Figure 2), with the MSN functionalized with positively charged aminopropyl groups and gated with negatively charged MUC1 aptamer via electrostatic and hydrogen bonding interactions (Pascual et al., 2017). Tumor uptake of ^99m^Tc-S1-apMUC1 was up to 20% ID/g with T/B ratio of about 7 at 2 h p. i. Notably, ^99m^Tc-S1-apMUC1 uptake in the liver and spleen was as low as about 1% ID/g, suggesting that ^99m^Tc-S1-apMUC1 nanoparticles bypassed elimination by the mononuclear phagocytic system (MPS). Its uptake in the lung was 15% ID/g, possibly due to the high-expression of MUC1 in this organ. High kidney uptake of about 20 %ID/g provides evidence of renal clearance. ^99m^Tc-S1-apMUC1 SPECT imaging can be a useful tool to detect MUC1 expression and predict the prognosis of MUC1-targeted treatment.

## Dual-Receptor Targeted Imaging of Breast Cancer

Recently, multiple antibodies, peptides, and nanoparticles have been developed to target two receptors simultaneously on the same cell or to elicit contact between two different cell types (Ehlerding et al., 2018). Those dual-receptor targeting strategies have multiple advantages over the mono-targeted ones such as improved target specificity and biodistribution *in vivo* (Kontermann, 2012). Most importantly, bispecific constructs targeting receptors expressed on the same cancer cell have the potential to overcome resistance mechanisms associated with mono-targeted therapies. Certain bispecific constructs have been designed so that they bind two distinct cells such as T cells and cancer cells and can therefore re-direct immune cells to tumor cells to stimulate cytotoxic activity (Ehlerding et al., 2018; Labrijn et al., 2019). The concept of dual-receptor targeting is of particular interest in heterogeneous subtypes of breast cancer, where a mono-targeted approach might fail to treat lesions absent of its target, especially in metastatic disease (McGuire et al., 2015; Peart, 2017). In this section, we discuss the progress made in the preclinical development and evaluation of bispecific agents with regards to imaging of breast cancer, independent of its molecular subtypes.

### GRPR/FA

Folate (FA) is a basic component of cell metabolism and DNA synthesis and repair. Folate receptor (FR) is a membrane-bound protein that binds and transports FA into cells (Frigerio et al., 2019) and its overexpression has been confirmed in all clinical breast cancer subtypes (Karuppaiah et al., 2020; Wang et al., 2020; Zhang et al., 2014b). As described above, overexpression of GRPR is also observed in 96% of all breast cancer tissues (Dalm et al., 2015). Therefore, an heterobivalent agent targeting GRPR and FR could improve breast cancer imaging. The radioconjugate ^99m^Tc-BBN-FA (Peptides, Figure 2) has been synthesized to target the GRPR with the BBN portion and the FR with the FA portion for SPECT imaging in preclinical models of breast cancer (Aranda-Lara et al., 2016b). Tumor uptake of ^99m^Tc-BBN-FA in T47D xenograft models was 5.4 ± 0.97% ID/g at 2 h p. i. and remained stable at 24 h p. i. with values of 2.5 ± 0.63% ID/g. Further, very fast blood clearance contributed to high T/B ratio of 124 at 24 h p. i. High uptake in the pancreas was observed due to the high GRPR expression in this organ. Tumor uptake of ^99m^Tc-BBN-FA was higher than that of ^99m^Tc-BBN or ^99m^Tc-FA alone, demonstrating the advantage of the bispecific construct. BBN and FA were also labeled with ^177^Lu (t_1/2_ = 6.71 days) for SPECT imaging in T47D xenograft models (Aranda-Lara et al., 2016a). ^177^Lu-BBN-FA (Peptides, Figure 2) showed similar excellent tumor uptake and biodistribution compared with the ^99m^Tc-labeled variant. In addition, when administering 74 MBq of each radiotracer, ^177^Lu-BBN-FA was shown to substantially enhance radiation absorbed dose in the tumor with up to 24 ± 2.1 Gy, as compared with those of the mono-targeted ^177^Lu-BBN and ^177^Lu-FA, which were lower by 47 and 67%, respectively. Clearly, the bispecific construct is also advantageous for therapy, as it can deliver greater radiation dose to the tumor. Taken together, these studies demonstrate that GRPR/FA dual-receptor targeted imaging perform better than its respective mono-specific variants and has potential for clinical translation for imaging and targeted radiotherapy of breast cancer.

### GRPR/NPY(Y_1_)R

A study conducted on human breast cancer patient tissues reported that 51% of them (32/63) showed an overexpression of GRPR together with another receptor called the neuropeptide Y receptor subtype 1 (NPY(Y_1_)R) (Reubi et al., 2002). Thus, a series of ^68^Ga-labeled heterobivalent peptidic ligands were synthesized to target both receptors with the goal of achieving increased binding to breast cancer cells over the mono-specific targeting agents (Vall-Sagarra et al., 2018). The best bispecific agent in this study was found to be the compound, ^68^Ga-24 (Peptides, Figure 2), with tumor uptake of 3.1 ± 0.33% ID/g and T/B ratio of 2.7 ± 0.43 at 130 min p. i. in the T47D xenograft models. Conversely, tumor uptake of the GRPR or NPY(Y_1_)R mono-specific targeted agents were lower, confirming the improved tumor uptake of the bispecific construct over the mono-targeted agents.

### α_v_β_3_/CD13

The integrin α_v_β_3_ receptor and CD13 are two other receptors whose expression levels are correlated with neoangiogenesis, invasiveness, metastasis, and poor overall survival in breast cancer (Ranogajec et al., 2012; Rolli et al., 2003). The ligands RGD and NGR bind to α_v_β_3_ and CD13, respectively, and have been used as anti-angiogenic drugs in radionuclide therapy (Debordeaux et al., 2018; Goodman and Picard, 2012). Hence, a bispecific agent derived from these mono-specific targeting agents, ^68^Ga-NGR-RGD (Peptides, Figure 2), has recently been synthesized for PET imaging in breast cancer xenografts (Gai et al., 2020). Tumor uptake in the MCF-7 xenograft models was 1.0 ± 0.16% ID/g with T/B ratio of about 6. Further, tumor uptake of ^68^Ga-NGR-RGD was significantly higher than that of ^68^Ga-NGR and ^68^Ga-RGD at 1 h p. i. More importantly, ^68^Ga-NGR-RGD detected lung metastases in MCF-7 xenografts. This study represents another proof of concept for the increased tumor targeting ability of bispecific agents over the mono-specific constructs.

### EGFR/HER2

As previously discussed, HER2 represents a common therapeutic target in the HER2 subtype of breast cancer. However, HER2-directed therapies such as trastuzumab can develop resistance through several mechanisms including heterodimerization of EGFR with HER2 (Dua et al., 2010). A ^64^Cu-labeled bispecific antibody fragment, ^64^Cu-NOTA-Fab-PEG_24_-EGF (Fab, Figure 2), was thus developed to inhibit the EGFR and HER2 receptors simultaneously (Kwon et al., 2017). In order to increase the blood circulation time and potentially increase tumor uptake of the tracer, Kwon et al. linked the Fab of the trastuzumab to that of the EGF through a PEG_24_ linker, conjugated the resulting construct to NOTA, and radiolabeled with ^64^Cu to obtain ^64^Cu-NOTA-Fab-PEG_24_-EGF. In the MDA-MB-231/H2N xenograft model, which is characterized by low expression of HER2 and moderate expression of EGFR, the bispecific ^64^Cu-NOTA-Fab-PEG_24_-EGF showed much greater tumor uptake (4.9% ID/g at 48 h p. i.) than those of the radiolabeled Fab (against HER2) and EGF monomers (1.9% ID/g and 0.7% ID/g, respectively). The highest uptake of ^64^Cu-NOTA-Fab-PEG_24_-EGF in normal organs was observed in the kidney with 25 ± 4.2% ID/g. Further investigation is needed to evaluate the ability of PET imaging with ^64^Cu-NOTA-Fab-PEG_24_-EGF to predict treatment response (efficacy) in HER2-and EGFR-directed therapies.

### T-Cell/CEA

Another bispecific agent is the AMG211, a T-cell engager antibody construct used in phase I trials for targeting carcinoembryonic antigen (CEA) (Kebenko et al., 2018; Pishvaian et al., 2016), an established therapeutic target in a number of solid tumors, including breast cancer (Tang et al., 2016, Wang et al., 2017). A PET companion diagnostic agent for AMG211 was recently developed by radiolabeling the antibody with ^89^Zr to obtain ^89^Zr-AMG211 (Antibodies, Figure 2) (Waaijer et al., 2018). Tumor uptake of ^89^Zr-AMG211 in CEA-positive BT-474 xenograft models was 3.8 ± 1.1% ID/g with T/B ratio of about 10 at 24 h p. i., while uptake was significantly lower in the CEA-negative HL-60 xenograft models (*p* < 0.01). A major drawback of this imaging agent is its extremely high uptake in the kidneys (∼150% ID/g).

### EGFR/C-MET

**FIGURE 5:**
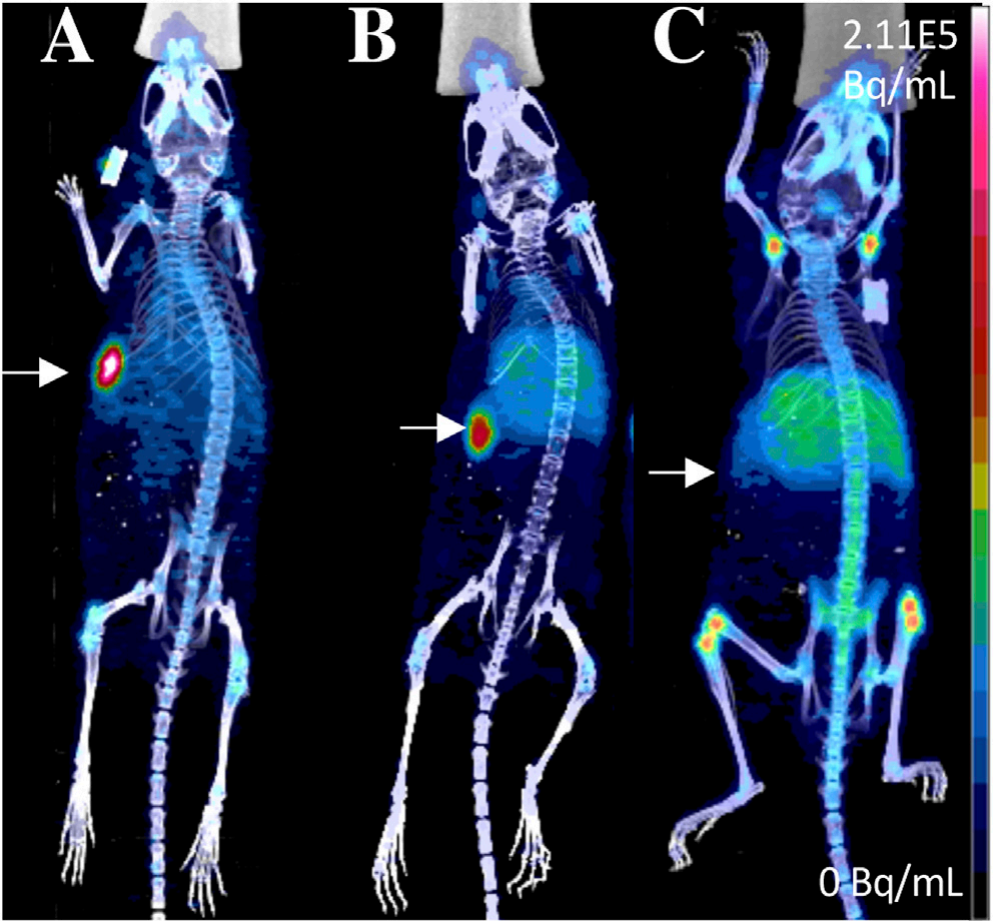
PET/CT imaging of [^89^Zr]ZrDFO-Amivantamab in MDA-MB-468 **(A)**, MDA-MB-231 **(B)** and MDA-MB-453 **(C)** xenografts of TNBC at 96 h p. i. Tumors are marked with arrows (Cavaliere et al., 2020). The general structure of [^89^Zr]ZrDFO-Amivantamab was shown in Figure 2H.

Amivantamab is a new bispecific antibody with multiple mechanisms of action, including inhibition of the EGFR and the hepatocyte growth factor receptor (HGFR/c-MET) pathways (Moores et al., 2016). In a recent report it was radiolabeled with ^89^Zr via a desferrioxamine chelate (DFO) to create a companion diagnostic imaging agent for this bispecific antibody (Cavaliere et al., 2020) (Bispecific antibodies, Figure 2). As overexpression of EGFR and c-MET was found in TNBC and associated with progression of the disease, the resulting [^89^Zr]ZrDFO-amivantamab radioconjugate was evaluated in TNBC xenograft models (Cavaliere et al., 2020; Chae et al., 2016). Three xenografts were used, MDA-MB-468, MDA-MB-231, and MDA-MB-453, which are characterized by high, moderate, and negative co-expression of EGFR and c-MET, respectively (Figure 5). PET/CT imaging with [^89^Zr]ZrDFO-amivantamab showed its ability to detect graded levels of EGFR and c-MET with standard uptake values (SUV_mean_) of 6.0 ± 1.1, 4.2 ± 1.4, 1.5 ± 1.4 96 h p. i. in MDA-MB-468, MDA-MB-231, and MDA-MB-453, respectively (Figure 5) (Cavaliere et al., 2020). Further, tumor uptake of [^89^Zr]ZrDFO-amivantamab was significantly higher than those of the radiolabeled single-arm parent antibodies [^89^Zr]ZrDFO-α-EGFR or [^89^Zr]ZrDFO-α-c-MET. This imaging agent has the potential to be clinically translated to provide a more quantitative assessment of the total expression of EGFR and c-MET for patient selection in clinical trials that evaluate the efficacy of amivantamab.

As more bispecific agents are being developed to overcome resistance and limitations associated with mono-targeted therapies, there is an increasing need for development of their companion diagnostic imaging agents. Molecular imaging has the potential to accelerate the development of novel bispecific constructs by predicting response and selecting patients most likely to benefit from these dual-targeted therapies.

## Preclinical Targeted Therapy Agents for Breast Cancer

The two strategies to incorporate cytotoxic payloads into targeting agents are to conjugate non-specific cytotoxic drugs, and to attach radioisotopes that emit DNA-damaging energy. These targeting agents typically employ peptides and antibodies, as they can be chemically modified with the cytotoxic payloads while maintaining their pharmacokinetic properties and specific binding to target proteins on the surface of cancer cells. These vehicles may internalize in the cancer cell once bound to the target protein and deliver their cytotoxic payload.

### Targeted Radionuclide Therapy

Targeted radionuclide therapy (TRT) combines the specificity of targeting molecules and the cytotoxicity of ionizing radiation as an approach to overcome resistance to other drugs (Gill et al., 2017). The diverse combinations of targeting molecules and radioisotopes provide flexible choices in the treatment of the different molecular subtypes of breast cancer for both primary and metastatic disease.

There are three types of radiation related to TRT: β-particles, α particles, and Auger electrons, which can irradiate volumes with multicellular, cellular, and subcellular dimensions, respectively (Gill et al., 2017). The β-emitters are considered ideal for targeting large tumors due to their long range path length of 0.05–12.0 mm in tissue, and the ability to induce formation of radical species that are damaging to DNA (Pouget et al., 2011). The α emitters, with a short-range path length of 20–100 μm, has a high linear energy transfer and are ideal for treating micrometastases and blood or bone marrow malignancies (Dahle et al., 2007). Finally, Auger electrons have the shortest range of 1–23 μm and are suitable for targeting single cells (Ku et al., 2019b).

Recently, ^177^Lu and ^111^In have attracted the most attention for TRT of breast cancer. ^177^Lu is a low-energy β-emitter (0.497 MeV_max_) with tissue penetration of up to 1.6 mm, which considerably lowers the dosimetry (i.e., radiation dose to organs and whole body) for patients (Massicano et al., 2018). The long half-life of ^177^Lu (6.71 days) also provides advantages in production and transportation to facilities that do not have the capability to produce this radioisotope. In contrast, ^111^In is an Auger electron with low-energy (<30 keV) and a very short path length of less than 10 μm, and must be delivered to the tumor cell nucleus to achieve maximum cell-killing ability via DNA double-strand breaks (Valkema et al., 2002; Boswell and Brechbiel, 2005). The radiopharmaceuticals based on Auger electrons can be enhanced by increasing nuclear localization, either by attaching a peptide with an nuclear localization sequence (NLS) (Costantini et al., 2010), or co-administer with other pharmaceuticals capable of intensifying nuclear localization (Bailey et al., 2007). In this section, we review examples of new antibody drug conjugates and TRT in breast cancer, as listed in Table 2.

**TABLE 2:** Preclinical targeted therapy agents for the different subtypes of breast cancer. Those with additional imaging properties are checked in the theranostics column.

Target	Agent	Structure	Imaging and therapy	Models	References
HER2-positive					
HER2	^89^Zr-AF-*Lx*-trastuzumab	Antibody conjugate	√	JIMT-1	Sijbrandi et al. (2017)
	^111^In-NLS-trastuzumab	Antibody-peptide conjugate	—	MDA-MB-361	Costantini et al. (2010)
	^111^In-trastuzumab-DOX-APTES-PEG-SPIONs	Nanoparticles, antibody and chemotherapeutics	√	SK-BR-3	Zolata et al. (2015)
HER2 and EGFR	^177^Lu-AuNPs-trastuzumab-panitumumab	Nanoparticle-antibody conjugate	—	MDA-MB-231-H2N; MDA-MB-468; BT-474	Yook et al. (2020)
**Triple negative**					
EGFR	^111^In-bn-DTPA-nimotuzumab	Antibody	—	MDA-MB-468	Chan et al. (2020)
PSMA	^177^Lu-PSMA-617/^68^Ga-PSMA-11	Small molecule	√	MDA-MB-231	Morgenroth et al. (2019)
**Subtype independent**					
GRPR	^177^Lu-DOTA-DN(PTX)-BN	Nanoparticle-peptide conjugate loaded with chemotherapeutics	√	T47D	Gibbens-Bandala et al. (2019a)
	^177^Lu-BN-PLGA(PTX)	Nanoparticle-peptide conjugate loaded with chemotherapeutics	√	MDA-MB-231	Gibbens-Bandala et al. (2019b)
FA	^99m^Tc-PEG-PAMAM G4-FA-5FU	Nanoparticle-peptide conjugate loaded with chemotherapeutics	√	MCF-7	Narmani et al. (2017)
Nucleolin	^111^In-BnDTPA-F3	Peptide	√	MDA-MB-231-H2N	Cornelissen et al. (2012)

### HER2-Positive Breast Cancer

Many kinds of HER2-directed agents have been labeled with different radionuclides and previously reviewed (Massicano et al., 2018). Here, we summarize recent strategies on the preclinical development of HER2-targeted therapy agents not discussed by Massicano et al.

Sijbrandi et al. used a novel strategy of conjugating an ethylenediamine platinum (*Lx*) to trastuzumab (Sijbrandi et al., 2017), with the expected effects of improved aqueous solubility for the *Lx*-payload complexes and the *Lx* able to coordinate to unique amino acids, including methionines, cysteines, and histidines, which is a valuable alternative to the currently used strategy of coupling to lysines and cysteines (Messori et al., 2014). In this study, auristatin F (AF) coordinated *Lx* was conjugated to trastuzumab and radiolabeled with ^89^Zr to obtain the companion diagnostic agent, ^89^Zr-AF-*Lx*-trastuzumab. The therapeutic efficacy of AF-*Lx*-trastuzumab was evaluated in HER2-positive and trastuzumab resistant JIMT-1 xenograft models. All tumors regressed completely with no regrowth observed until the end of the experiment at day 125, indicating that all xenografted tumors had complete response in mice treated with AF-*Lx*-trastuzumab. In contrast, only 25% of mice had complete response when treated with the ado-trastuzumab emtansine (T-DM1) control. The therapeutic efficacy of AF-*Lx*-trastuzumab demonstrates its superiority over the T-DM1 standard-of-care. While these results are promising, toxicity studies in higher species are needed before translation to clinical evaluation.

An ^111^In-labeled trastuzumab was modified with the nuclear localization sequence (NLS) peptides (CGYGPKKKRKVGG) to obtain ^111^In-NLS-trastuzumab (Costantini et al., 2010). Tumor growth was delayed in the HER2-positive MDA-MB-361 xenografts treated with a single dose of ^111^In-NLS-trastuzumab (9.25 MBq, 4 mg/kg). On the contrary, ^111^In-NLS-trastuzumab had no effect on tumor growth of the HER2-negative MDA-MB-231 xenografts. When two doses (9.25 MBq, 4 mg/kg) of ^111^In-NLS-trastuzumab were administered two weeks apart, the survival time of MDA-MB-361 xenograft models was significantly prolonged and 50% of the tumors (3 of 6 mice) regressed completely. Based on these results, ^111^In-NLS-trastuzumab achieved high targeted radiotherapeutic efficacy in HER2-positive tumors.

Nanotechnology represents a hot area in drug delivery research. Superparamagnetic iron oxide nanoparticles (SPIONs) with appropriate surface modification have been widely used for biomedical applications. For example, SPIONs decorated with trastuzumab-doxorubicin (DOX) conjugate and labeled with ^111^In were evaluated as a theranostic agent in HER2-positive SK-BR-3 xenograft models (Zolata et al., 2015). Tumor uptake of the ^111^In-labeled SPIONs was 13 ± 0.76% ID/g with T/B ratio of 10 at 48 h p. i. After treatment with ^111^In-labeled trastuzumab-DOX conjugated SPIONs, tumor volumes were reduced by 36% in 3 weeks, while the tumor volumes of the control group were 4-fold larger than those in the treated group. Therapeutic efficacy was increased due to appropriate surface modification on SPIONs to prolong circulation time, specific targeting by trastuzumab, controlled DOX release, and Auger electrons and gamma rays of the ^111^In radionuclide.

Recent studies reported that trastuzumab resistance in HER2-positive cells might be due to activation of the EGFR pathway and hence increased EGFR protein expression. The heterodimers formed between EGFR and HER2 may circumvent the anti-tumor effects of HER2-targeted therapies. A bispecific agent ^177^Lu-AuNPs-trastuzumab-panitumumab was developed to overcome resistance to trastuzumab by targeting both HER2 and EGFR simultaneously (Yook et al., 2020). This dual-receptor-targeted agent was specifically bound and internalized by breast cancer cells that expressed HER2, or EGFR, or both, and showed high absorbed radiation doses with 36–119 Gy in the cell nucleus treated with ^177^Lu-AuNPs-trastuzumab-panitumumab. Although the study was conducted *in vitro*, this agent is promising for further evaluation *in vivo* in breast cancer xenografts.

### Triple-Negative Breast Cancer

One potential strategy to overcome drug resistance in TNBC is to combine mAbs with therapeutic radionuclides. Nimotuzumab is a mAb that binds to EGFR and clinically used in several countries for the treatment of epithelial-derived tumors that overexpress EGFR (Mazorra et al., 2018). In one recent study, ^111^In-Bn-DTPA-nimotuzumab (Antibodies, Figure 2) was prepared by conjugating nimotuzumab to benzyl isothiocyanate DTPA (Bn-DTPA) and radiolabeling with ^111^In, and evaluated in MDA-MB-468 xenograft models (Chan et al., 2020). Therapeutic efficacy was demonstrated by its enhanced inhibition of tumor growth, where the tumor doubling ratio of MDA-MB-468 xenografts was about 2-fold longer than those treated with the unlabeled Bn-DTPA-nimotuzumab or saline. ^111^In-Bn-DTPA-nimotuzumab may provide an alternative strategy for targeted treatment of TNBC. This approach might be beneficial to the basal-like subtype of TNBC, whose gene expression profiles suggest sensitivity to therapies that employ DNA damage mechanisms (Lehmann et al., 2011).

**FIGURE 6:**
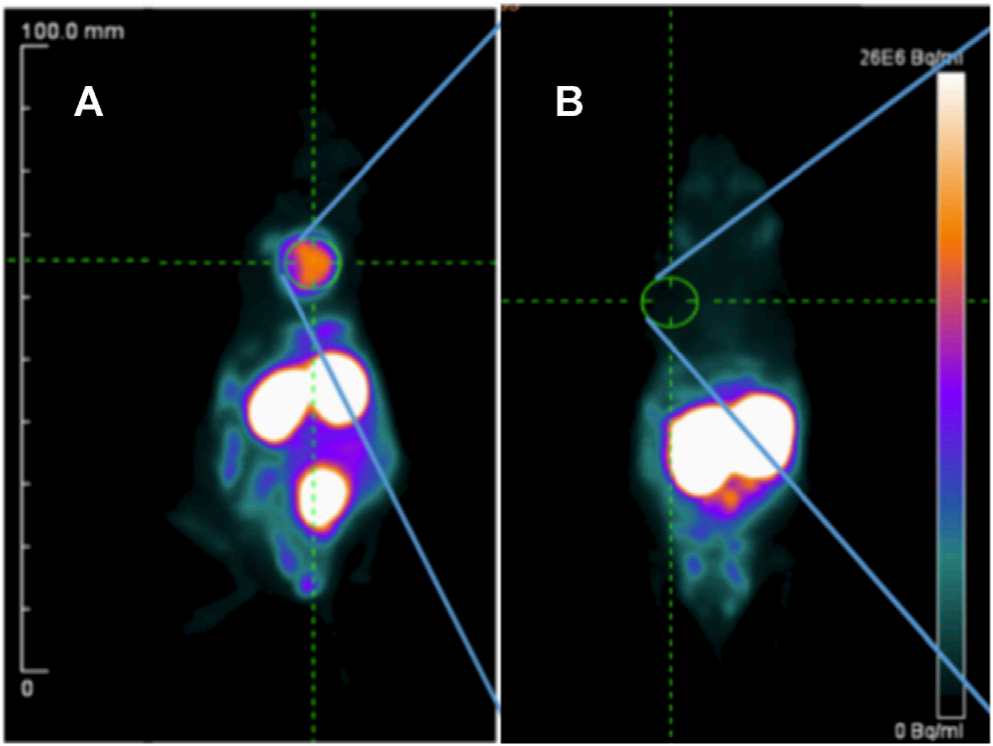
PET imaging of ^68^Ga-PSMA-11 in MDA-MB-231 **(A)** and MCF-7 **(B)** xenografts at 30 min post-injection (Morgenroth et al., 2019).

Prostate-specific membrane antigen (PSMA) is an established target for theranostics of prostate cancer, but a potential new target for breast cancer. A recent study reported that PSMA was expressed in tumor cells and tumor-associated neovasculature of primary breast cancer and distant metastases, while normal breast tissues expressed PSMA only in the glandular cells (Kasoha et al., 2017). One recent study evaluated the efficacy of radiolabeled PSMA-ligand in TNBC models (Morgenroth et al., 2019). High specific tumor uptake of ^68^Ga-PSMA-11 was shown in MDA-MB-231 xenografts with T/B ratio of 43.3 ± 0.9 at 30 min p. i., while tumor uptake in the control MCF-7 xenografts was negligible, with T/B ratio of 1.1 ± 0.1 (Figure 6). The MDA-MB-231 cells showed a high pro-angiogenic potential on tube formation of endothelial huvec cells. ^177^Lu-PSMA-617 strongly impaired the vitality and angiogenic potential of MDA-MB-231 medium-conditioned HUVEC cells. This study presented the rationale for PSMA-targeted therapy for TNBC.

### Subtype-Independent Therapy Agents

**FIGURE 7:**
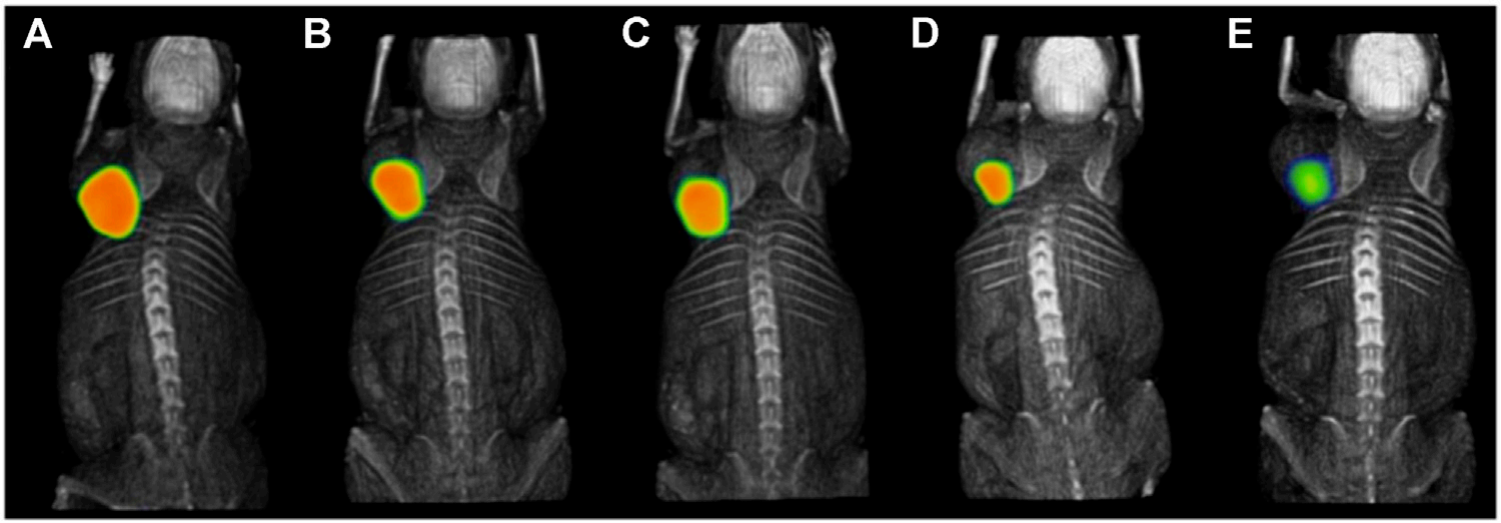
Intratumoral administration of ^177^Lu-DOTA-DN(PTX)-BN after 1.5 h **(A)**, 9 h **(B)**, 10 h **(C)**, 24 h **(D)**, and 120 h **(E)** in T47D xenograft model (Gibbens-Bandala et al., 2019a). The general structure of ^177^Lu-DOTA-DN(PTX)-BN was shown in Figure 2I.

The peptide-receptor radionuclide therapy (PRRT) is an approach that uses radiolabeled peptides that bind to receptors on the surface of cancer cells for specific delivery of ionizing radiation. Since GRPR are overexpressed across all subtypes of breast cancer, PRRT of GRPR might have a more general application for breast cancer treatment. To increase the stability of the targeting peptide, nanoparticles have been increasingly used as drug delivery vehicles. A nanosystem based on the ^177^Lu-labeled polyamidoamine (PAMAM) dendrimer (DN) loaded with paclitaxel (PTX) and functionalized on the surface with the DOTA-BBN peptide was designed for specific targeting to GRPR in T47D breast cancer xenografts (Gibbens-Bandala et al., 2019a). The ^177^Lu-DOTA-DN(PTX)-BBN nanoconjugate had significant uptake and internalization in T47D cells, with an estimated absorbed radiation dose of 3.0 Gy/MBq at infinite time. Tumor uptake of ^177^Lu-DOTA-DN(PTX)-BBN was about 35% ID/g at 120 h p. i., with a corresponding reduction in tumor volume by 16% (Figure 7). Another nanoparticle, poly lactic-co-glycolic acid (PLGA), was also evaluated for delivery of drugs and radiation (Gibbens-Bandala et al., 2019b). A PTX-loaded PLGA was conjugated to DOTA-BBN, labeled with ^177^Lu, and tested in MDA-MB-231 xenografts. The ^177^Lu-BBN-PLGA (PTX) treated group showed the lowest tumor proliferation and strongest inhibition of tumor growth among the other control groups. The average absorbed radiation dose in the tumor was 37 ± 7.0 Gy. These two nanosystems both exhibited enhanced therapeutic efficacy due to β-radiation from ^177^Lu and controlled release of PTX. Another example of ^177^Lu-labeled nanosystem is a dendrimer conjugated to folate and BBN with gold nanoparticles in the dendritic cavity (Mendoza-Nava et al., 2017). The bispecific ^177^Lu-DenAuNP-FA-BBN showed high absorbed radiation dose with 63 ± 4.2 Gy delivered to T47D cells. Further studies are needed to evaluate its therapeutic efficacy *in vivo*. Taken together, these strategies of using ^177^Lu-labeled chemotherapeutic drug-loaded nanosystems with BBN peptides for combined targeted therapy have shown promise in their application to GRPR-positive breast cancers.

Besides the classic breast cancer targets, such as ER, PR, HER2, EGFR, and recently, GRPR, other targets such as FA and nucleolin have also shown some promise for theranostics of breast cancer. A drug delivery nanosystem based on ^99m^Tc-labeled 5-fluorouracil (5-FU)-loaded and FA-functionalized PAMAM G4 dendrimer (^99m^Tc-PEG-PAMAM G4-FA-5FU) was designed for chemotherapy delivery to FA-overexpressing tumors with ^99m^Tc serving as the SPECT reporter for this treatment, and high tumor uptake of the agent in MCF-7 xenografts (Narmani et al., 2017).

An ^111^In-labeled F3 peptide (Peptides, Figure 2), which is a fragment of the human high mobility group protein 2-binding nucleolin, was developed to investigate the Auger electron-targeted radiotherapy in HER2-positive breast cancer xenograft models (Cornelissen et al., 2012). Animal treated with ^111^In-BnDTPA-F3 showed significantly slower tumor growth and longer survival time. SPECT imaging is feasible with this agent.

## Discussion and Perspectives

In the era of precision medicine, theranostic agents are becoming increasingly important for selecting breast cancer patients most likely to benefit from targeted treatments through imaging and offering more options for effective treatments in this heterogenous disease. In this review, we highlight many imaging probes for novel targets with potential for translation to clinical studies. Among the different targets discussed for breast cancer imaging, the GRPR is the most promising novel target in our opinion. GRPR is reported to be overexpressed in 96% of breast cancer tissues across all molecular subtypes of breast cancer. The GRPR-targeted agents for both imaging and therapy achieved excellent tumor uptake, such as ^111^In-JMV4168 for luminal-subtype imaging (Dalm et al., 2015), ^68^Ga-NOTA-PEG_3_-RM26 for HER2-subtype imaging (Varasteh et al., 2014), ^99m^Tc-BN4 for TNBC imaging (De et al., 2019), ^177^Lu-DOTA-DN(PTX)-BN for luminal-subtype therapy (Gibbens-Bandala et al., 2019a), and ^177^Lu-BN-PLGA (PTX) for TNBC therapy (Gibbens-Bandala et al., 2019b). These independent studies indicate that GRPR is a highly promising target for theranostics of breast cancer.

Additionally, there are many targeted small molecule inhibitors under clinical trials for the treatment of breast cancer, such as the CDK4/6 inhibitor palbociclib (Song et al., 2019; Gan et al., 2020; Ramos et al., 2020), the PI3K/Akt/mTOR pathway inhibitor pictilisib (Altine et al., 2019; Han et al., 2019), the HDAC inhibitor CUDC-101 (Meng et al., 2013). The radiolabeled analogs of these small molecule inhibitors that we described in this review showed high tumor uptake in breast cancer xenografts. These agents may provide a non-invasive diagnostic imaging tool to monitor the responses to their therapeutic equivalents.

Recently, dual-receptor targeted strategies have attracted increasing attention in heterogeneous subtypes of breast cancer imaging within primary and metastatic lesions. One of the reasons for their success is that bispecific constructs targeting two receptors can help to overcome drug resistance associated with mono-targeted therapies (Thakur et al., 2018). Different bispecific imaging and/or theranostic agents have also been developed, notably the scaffolds targeting GRPR/FA and EGFR/c-MET with ^99m^Tc/^177^Lu-BBN-FA (Aranda-Lara et al., 2016a; Aranda-Lara et al., 2016b) and [^89^Zr]ZrDFO-amivantamab (Cavaliere et al., 2020), respectively. These agents hold potential for clinical translation due to the high expression of targets in several molecular subtypes of breast cancer and the promise to overcome resistance to mono-targeted therapy due to their multiple mechanisms of action. Overall, a significant progress has been made in pursing novel targets for breast cancer imaging.

Although the studies mentioned above have shown promising results in rodent xenografts and in the setting of primary breast cancer, most of them did not focus on metastatic models. Of all the studies we reviewed, only ^68^Ga-NGR-RGD was evaluated in a lung metastasis model of breast cancer (Gai et al., 2020). The most typical sites of metastatic breast cancer are regional lymph nodes, bone, liver, lung, and brain (Jin et al., 2018). Hence, a good imaging agent should reach these organs, but there are still many challenges to overcome. For instance, a high liver uptake, commonly seen in PET and SPECT imaging with tracers that are metabolized in the liver, such as ^99m^Tc-DTPA-estradiol (Xia et al., 2016), ^99m^Tc-labeled palbociclib analogs (^99m^Tc-L2 to L5) (Song et al., 2019), and ^11^C-pictilisib (Han et al., 2019), may be insensitive in detecting liver metastasis. Thus, future studies should make more efforts into evaluating imaging agents in the metastatic setting.

Crossing the blood-brain barrier (BBB) represents another dilemma for detecting brain metastasis. The BBB with its tight junctions limits the passage of large molecules from the blood to the brain (Deeken and Löscher, 2007). In addition, there are various efflux transporters expressed in the BBB, including P-glycoprotein and breast cancer resistance protein, which contribute to restrict the entry of potentially therapeutic agents (de Vries et al., 2007). A recent study reported that trastuzumab conjugated with melanotransferrin may help treat brain metastasis, and melanotransferrin may be a potential delivery vehicle to increase antibody transport across the BBB (Nounou et al., 2016).

A limitation of the preclinical studies we introduced above is that they all used animal xenograft models due to poor or no cross-reactivity to mouse antigens. While these animal models allow for a convenient method to determine specificity *in vivo* for human targets, they do not capture accurate biodistribution to normal organs, which pose challenges with using rodents for dosimetry estimates for clinical translation. Nevertheless, companion imaging agents such as those described in this review have the potential to predict and monitor response to treatment, especially for the diverse molecular subtypes in breast cancer. The preclinical studies described in this review showed promising results of targeted imaging in breast cancer xenografts. Validation in human studies warrants further investigation.

With regard to TRT, ^177^Lu is a widely used radionuclide due to its relatively long-range in tissues (Massicano et al., 2018), which allows a cross-fire effect with the surrounding cells into the tumor. ^111^In can exhibit high therapeutic efficacy after being delivered to the tumor cell nucleus to maximize the cell-killing ability with the methods of attaching nuclear localization sequence (NLS) peptides, such as ^111^In-NLS-trastuzumab (Costantini et al., 2010), and co-administration with other pharmaceuticals including mAbs such as ^111^In-Bn-DTPA-nimotuzumab (Chan et al., 2020).

Another useful tool for drug delivery is nanosystems, especially to deliver chemotherapeutic drugs, mAbs, or their combination, such as ^111^In-trastuzumab-DOX-SPIONs (Zolata et al., 2015), ^177^Lu-AuNPs-trastuzumab-panitumumab (Yook et al., 2020), and ^177^Lu-DOTA-DN(PTX)-BBN (Gibbens-Bandala et al., 2019a). TRT combined with chemotherapy or antibodies have proven to be beneficial to breast cancer treatment. Although the tumor uptake and absorbed radiation dose might vary considerably between different patients, precision medicine for breast cancer patients may help to make TRT more effective and reduce normal tissue toxicity.

## Conclusion

Targeted imaging and therapy using nuclear medicine methods show promise for precision medicine for patients with breast cancer. Molecular imaging can help with diagnosis, staging, guiding treatment, and predicting response to corresponding targeted therapy. Many studies discussed here have made great contributions in the investigation of new strategies and new agents for breast cancer imaging and therapy. A series of new targets have been found to be valuable for potentially overcoming resistance to standard of care treatments. These new investigations are inspiring in preclinical studies. We look forward to seeing more studies advance to clinical trials in the near future.

## Data Availability

The original contributions presented in the study are included in the article/Supplementary Material, further inquiries can be directed to the corresponding author.
